# Launch of *IJTLD OPEN*: a new home for open access papers on respiratory disease

**DOI:** 10.5588/ijtldopen.23.0598

**Published:** 2024-01-01

**Authors:** H. D. Blackbourn, G. B. Migliori

**Affiliations:** ^1^ International Union Against Tuberculosis and Lung Disease, Paris, France;; ^2^ Istituti Clinici Scientifici Maugeri, Istituto di Ricovero e Cura a Carattere Scientifico, Tradate, Italy

**Keywords:** open access, *International Journal of Tuberculosis and Lung Disease*, The Union, cOAlition S

The *International Journal of Tuberculosis and Lung Disease* (*IJTLD*) has traditionally operated as a hybrid journal, with subscription content and open access (OA) articles.^1^ However, the members of cOAlition S have mandated their authors to publish exclusively in OA journals, and hybrid journals are no longer supported. Given that a key part of the Union’s mission is to improve the dissemination of knowledge, we are keen to support and embrace this move to OA. It is therefore our pleasure to welcome you to this first issue of *IJTLD OPEN*, a fully OA journal.

In 2011, the late Donald Enarson wrote a similar introductory Editorial in the inaugural issue of Public Health Action (PHA).^2^ As usual, Don was ahead of his time and PHA was an early adopter of OA. He commented, ‘When we consider the burden of disease the poor suffer, we must conclude that relieving this burden is not primarily in knowing what to do, but rather in knowing how to do it.’ *IJTLD OPEN* will continue to explore this question of ‘what do to’ to improve lung health, but also to focus on the ‘how’ and ‘when’ for clinicians to reduce the incidence and severity of respiratory disease.

Scientific publishing is currently in a state of flux with the launch of many OA journals. Regrettably, some of these have gained a reputation for poor quality. To protect against this, we have adopted exactly the same values and scope for *IJTLD OPEN* as the *IJTLD*, and the peer review process and acceptance criteria are identical.^1^ In this way, articles are selected for publication without regard for funding source, and it is only after acceptance that they diverge into either the *IJTLD* or *IJTLD OPEN*. We believe this approach, which preserves the scientific integrity of the subject matter while allowing us to adapt to new funding mechanisms, will also improve access to scientific knowledge.

For our readers, there are significant advantages to OA: there are no barriers to access, so anyone, anywhere can download an article to read for free. This will dramatically increase our readership, particularly for people living in low- to middle-income countries (LMICs) where the cost of a subscription may be prohibitive, but which typically have the highest burden of disease. Authors will also benefit from this increased visibility: instead of only being visible to a relatively small number of subscribers, the articles are available to all. Also, according to the traditional subscription model, the publishers retained copyright and authors had to ask permission to reuse their own work. For OA, we return to a more equitable situation where authors retain copyright and can use the article in any way they see fit – either in sending a PDF to their colleagues or for use in teaching. There are no restrictions whatsoever.

This first issue of *IJTLD OPEN* features a representative collection of articles, proportionate to the level of OA funding available for TB and respiratory disease in LMICs. This includes Editorials highlighting TB in children and adolescents,^3^ and the need for action after the UN High-Level Meeting on TB.^4^ The mix of Original Articles includes ‘Characteristics of TPT initiation and completion among people living with HIV’,^5^ ‘Screening of household contacts for TB infection’,^6^ ‘Clinical spectrum of disease and outcomes in children with Omicron SARS-COV-2 infection’,^7^ ‘Key drivers of the TB epidemic in Suriname’,^8^ ‘Timeliness metrics for screening and preventing TB in household contacts of pulmonary TB patients’,^9^ and ‘Commitment, partnerships and operational research: three priorities for 11 EMR countries to achieve TB elimination’.^10^

We believe that OA is a highly effective and equitable publishing model, which will drive the dissemination of knowledge for future generations. Our hope and expectations are that *IJTLD OPEN* will play a significant role in this process. Instead of restricting access to subscribers, OA allows everyone to access the content, which will ensure that the *Journal* is widely read and cited. This is the pattern we have seen to date, with OA articles in the IJTLD typically having the highest number of downloads and citations.^11–13^ OA also has societal benefits, with improved awareness and understanding of respiratory disease for all concerned. In this way, we can address issues such as stigma, and promote greater access to treatment, helping to remove barriers to improved healthcare.
